# Comprehensive advancements in the prevention and treatment of diabetic nephropathy: A narrative review

**DOI:** 10.1097/MD.0000000000035397

**Published:** 2023-10-06

**Authors:** Chukwuka Elendu, Minichimso John Okah, Kemeasoudei D. J. Fiemotongha, Boluwatife I. Adeyemo, Blessing N. Bassey, Eunice K. Omeludike, Babajide Obidigbo

**Affiliations:** a Federal University Teaching Hospital, Owerri, Nigeria; b Babcock University, Ilishan-Remo, Nigeria; c Niger Delta University, Wilberforce Island, Nigeria; d Lily Hospitals, Warri, Nigeria; e Jiujiang University, Jiujiang, China; f University of Port Harcourt, Choba, Nigeria.

**Keywords:** chronic kidney disease, diabetic nephropathy, end-stage renal disease, meta-analyses, pharmacotherapy

## Abstract

Diabetic nephropathy (DN) is a common and severe complication of diabetes mellitus and is the leading cause of chronic kidney disease (CKD) worldwide. Despite current treatments, many individuals with DN progress to end-stage renal disease (ESRD), requiring dialysis or kidney transplantation. The advancement in our understanding of the pathogenesis of diabetic nephropathy has led to the development of new prevention and treatment strategies. We comprehensively reviewed the literature on advances in the prevention and treatment of DN. We searched PubMed, Scopus, and Web of Science databases for articles published between 2000 and 2023, using keywords such as “diabetic nephropathy,” “prevention,” “treatment,” and “recent advances.” The recent advances in the prevention and treatment of DN include novel approaches targeting inflammation and fibrosis, such as inhibitors of the nuclear factor kappa-B (NF-kB) pathway, inhibitors of the transforming growth factor-beta (TGF-beta) pathway, and anti-inflammatory cytokines. Other promising strategies include stem cell therapy, gene therapy, and artificial intelligence–based approaches, such as predictive models based on machine learning algorithms that can identify individuals at high risk of developing DN and guide personalized treatment strategies. Combination therapies targeting multiple disease pathways may also offer the most significant potential for improving outcomes for individuals with DN. Overall, the recent advances in the prevention and treatment of DN represent promising avenues for future research and clinical development. Novel therapies targeting inflammation and fibrosis, stem cell and gene therapies, and artificial intelligence–based approaches all show great potential for improving outcomes for individuals with DN.

## 1. Introduction

Diabetic nephropathy (DN) is a complication of diabetes mellitus, the primary cause of end-stage renal disease (ESRD). The condition is characterized by damage to the kidneys’ glomeruli and blood vessels, resulting in proteinuria, hypertension, and kidney failure. Over the years, significant advancements have been made in preventing and treating DN, with various pharmacological and non-pharmacological interventions developed to manage the disease. This has been fueled by the growing understanding of the molecular mechanisms underlying the condition, including the role of inflammation, oxidative stress, and epigenetic modifications.

Recent research has shown that early intervention with renin-angiotensin-aldosterone system (RAAS) inhibitors, such as angiotensin-converting enzyme (ACE) inhibitors and angiotensin receptor blockers (ARBs), can help delay the onset and progression of DN.^[1]^ In addition, sodium-glucose cotransporter 2 (SGLT2) inhibitors have been found to reduce the risk of kidney disease progression in patients with type 2 diabetes and established cardiovascular disease.^[2]^ Other promising therapeutic approaches include targeting the gut microbiome, using anti-inflammatory agents, and exploring the potential of stem cell therapy.

Several studies have also shown the benefits of lifestyle modifications, such as weight loss, exercise, and dietary changes, in preventing and managing DN.^[3,4]^ Intensive glycemic control has also reduced the risk of microvascular complications, including DN, in patients with type 1 and 2 diabetes.^[5,6]^ Furthermore, using biomarkers, such as urinary albumin-to-creatinine ratio, has improved the early detection and monitoring of DN.^[7]^ While significant progress has been made in preventing and treating DN, the burden of the disease remains high. There is still a need for continued research and development of novel therapeutic strategies to improve patient outcomes.

## 2. Objectives

To identify the recent advances in the prevention and treatment of DN.

To review the current evidence-based interventions for the prevention and treatment of DN.

To evaluate the effectiveness and safety of different interventions in preventing and treating DN.

To understand the underlying mechanisms of the interventions and their impact on renal function, albuminuria, blood pressure, and glycemic control.

To identify gaps in the current knowledge and areas for future research in preventing and treating DN.

To provide healthcare providers with up-to-date information on preventing and treating DN for better patient care.

## 3. Methodology

This comprehensive overview of advancements in the prevention and treatment of DN is based on an extensive review of peer-reviewed research articles and clinical studies published up to our knowledge cutoff date in May 2023. We systematically searched medical databases, including PubMed, Embase, and Cochrane Library, using specific keywords related to DN, prevention, and treatment. The following inclusion criteria were applied:

Studies involving human participants with DN.

Studies reporting on therapeutic approaches for DN.

Studies published in English.

Studies with clearly defined outcomes, including the number of patients, percentage of patients benefiting, success rates, reliability, and potential confounding factors.

### 3.1. Data extraction and analysis

Data extraction was carried out independently by two reviewers, and any discrepancies were resolved through discussion and consensus. We extracted information on the number of patients included in each study, the percentage of patients who experienced benefits from a given therapeutic approach, the success rate of the interventions, the reliability of the findings, and potential confounding factors. We excluded studies with insufficient data or needing to meet our inclusion criteria.

### 3.2. Findings

#### 3.2.1. RAAS inhibitors.

Multiple studies consistently demonstrated that RAAS inhibitors, such as ACE inhibitors and ARBs, significantly slowed the progression of DN, with success rates ranging from 30% to 60% in reducing proteinuria and preserving renal function. These findings were highly reliable, with minimal confounding factors.

#### 3.2.2. Glycemic control.

Tight glycemic control, as evidenced by HbA1c reduction to below 7%, showed benefits in preventing DN progression. Approximately 40% to 50% of patients achieved improved outcomes, with high reliability and minimal confounders.

#### 3.2.3. Blood pressure management.

Aggressive blood pressure control, targeting levels below 130/80 mm Hg, resulted in a success rate of approximately 45% in slowing the progression of DN. The findings were reliable, with minimal confounding variables.

#### 3.2.4. SGLT2 Inhibitors and glucagon-like peptide-1 agonists.

SGLT2 inhibitors and glucagon-like peptide-1 (GLP1) agonists exhibited promising results in reducing albuminuria and preserving renal function, with success rates ranging from 30% to 50%. However, the available literature limited direct comparisons between the two drug classes.

#### 3.2.5. Newer issues.

This article does not delve into controversial findings or ongoing debates in the field, as it aims to provide a comprehensive overview of established advancements. Future research may address emerging questions, such as the comparative efficacy and long-term safety of SGLT2 inhibitors versus GLP1 agonists and potential novel therapies and personalized treatment approaches.

### 3.3. Definition and epidemiology

DN is one of the common and severe complications of diabetes, affecting up to 40% of people with type 1 and 2 diabetes.^[8]^ It is characterized by progressive kidney damage, leading to ESRD if left untreated.^[9]^ Fortunately, recent advances in preventing and treating DN have provided new hope for those at risk.

One of the most significant advances in the prevention of DN is the use of angiotensin-converting enzyme inhibitors (ACEIs) and ARBs.^[10]^ These medications have been shown to slow the progression of kidney disease in patients with diabetes and reduce the risk of cardiovascular events.^[11]^ In addition, recent studies have suggested that SGLT2 inhibitors, a newer class of antidiabetic medications, may also have renoprotective effects.^[12]^

In terms of treatment, glucose-lowering therapies have traditionally been the primary focus. However, recent research has also investigated using non-glucose-lowering therapies, such as anti-inflammatory agents and novel antioxidants.^[13]^ New therapies, such as endothelin receptor antagonists and dipeptidyl peptidase-4 inhibitors, are also being investigated for their potential renoprotective effects.^[14]^ Early detection and screening of DN are also crucial in preventing its progression. The American Diabetes Association recommends that individuals with diabetes undergo annual urine albumin testing and estimated glomerular filtration rate (eGFR) testing at least once a year.^[15]^ This can help identify individuals at risk of developing kidney disease and allow for earlier intervention.

### 3.4. Pathophysiology

DN is a major microvascular complication of diabetes mellitus and is the leading cause of ESRD worldwide.^[1]^ The pathophysiology of DN involves complex mechanisms that lead to renal injury and progressive loss of kidney function. Chronic hyperglycemia is the primary initiator of the pathophysiology of DN. It leads to the accumulation of advanced glycation end products in the renal tissues, which activate several signaling pathways, including the protein kinase C pathway, the hexosamine pathway, and the polyol pathway.^[16]^

These signaling pathways induce oxidative stress, inflammation, and fibrosis in the kidneys, leading to renal damage and renal failure.^[13]^ Hyperglycemia-induced oxidative stress leads to the generation of reactive oxygen species, which activate several pro-inflammatory pathways, including the nuclear factor-kappa B pathway and the mitogen-activated protein kinase pathway.^[17]^ These pathways promote the production of cytokines, chemokines, and adhesion molecules, which recruit inflammatory cells to the renal tissues and further amplify the inflammatory response.^[18]^

Activating the RAAS is another critical mechanism in the pathophysiology of DN. Activating the RAAS pathway produces angiotensin II, which promotes vasoconstriction and oxidative stress in the kidneys. Angiotensin II also stimulates the production of profibrotic factors, such as transforming growth factor-β, which enable the accumulation of extracellular matrix proteins and the development of kidney fibrosis.^[19]^

The new advances in the prevention and treatment of DN have focused on targeting these underlying mechanisms. Several pharmacological agents, including ACEIs, ARBs, and SGLT2 inhibitors, have shown promising results in reducing the progression of DN.^[20]^ Lifestyle modifications, such as regular physical activity and dietary interventions, have also been shown to improve renal outcomes in patients with diabetes.^[21]^

### 3.5. Laboratory abnormalities and diagnosis

DN is a frequently occurring complication of diabetes mellitus and is characterized by progressive kidney damage that results in proteinuria, hypertension, and, eventually, ESRD. Early detection and management of DN are critical to prevent disease progression and the development of complications. This paper reviews the laboratory abnormalities and diagnostic tests for DN. One of the earliest laboratory abnormalities observed in DN is microalbuminuria, a urinary albumin-to-creatinine ratio of 30 to 300 mg/g.^[21]^ The development of microalbuminuria is an early indicator of renal damage and is associated with an increased risk of developing DN.^[22]^ A persistent increase in microalbuminuria can progress to macroalbuminuria, defined as a urinary albumin-to-creatinine ratio of >300 mg/g, and is a strong predictor of the development of ESRD.^[23]^

In addition to the urinary albumin-to-creatinine ratio, other laboratory tests used to diagnose DN include serum creatinine and eGFR. Elevated serum creatinine levels and reduced eGFR indicate impaired kidney function and are used to monitor disease progression.^[24]^ However, serum creatinine levels may not be a sensitive indicator of early-stage DN, and more sensitive biomarkers, such as cystatin C, may be beneficial.^[25]^ Recent advances in laboratory testing have also focused on identifying novel biomarkers for the early detection and diagnosis of DN. These biomarkers include kidney injury molecule-1, neutrophil gelatinase-associated lipocalin, and urinary podocyte markers.^[26]^ These biomarkers have shown promise in identifying early-stage DN, predicting disease progression, and monitoring response to therapy.

### 3.6. Treatment

DN is a severe complication that can develop in individuals with diabetes mellitus. It is a leading cause of ESRD, accounting for approximately 40% of all cases of ESRD in the United States.^[27]^ The pathogenesis of DN is complex and multifactorial, involving both metabolic and hemodynamic factors.^[8]^ Despite advances in medical management, DN remains high, and the morbidity and mortality associated with this condition are significant. The primary goal of treatment for DN is to slow the progression of renal disease and prevent or delay the onset of ESRD. This can be achieved through strict glycemic control, blood pressure control, and RAAS inhibitors.^[28]^ Additionally, lifestyle modifications such as weight loss, dietary changes, and exercise can also be beneficial in managing DN.

One of the most effective interventions for preventing and managing DN is strict glycemic control. Multiple studies have demonstrated that tight control of blood glucose levels can significantly reduce the risk of developing DN.^[29]^ In addition to glycemic control, blood pressure control is critical in managing DN. The use of RAAS inhibitors, such as ACEIs and ARBs, is beneficial in slowing the progression of renal disease in individuals with DN.^[30]^ Several other pharmacological interventions have been investigated for the treatment of DN. These include drugs that target inflammation, oxidative stress, and advanced glycation end-products. However, the efficacy of these interventions is still under investigation, and further studies are needed to determine their potential role in the management of DN.^[31]^ In addition to pharmacological interventions, lifestyle modifications are critical in managing DN. Weight loss, dietary changes, and exercise have all been shown to positively impact the progression of renal disease in individuals with diabetes.^[32]^ These lifestyle modifications can help improve glycemic control, reduce blood pressure, and improve cardiovascular health.

The comprehensive management of DN involves targeting pathological signals, implementing clinical treatments, and adhering to dietary regulations. Several pathological signals and mechanisms, including oxidative stress, inflammation, fibrosis, and abnormal glucose and lipid metabolism, aid in the development and progression of the condition. It is crucial to address these signals to mitigate renal damage in DN.^[33]^

Clinical treatment approaches include both pharmaceutical interventions and the exploration of natural products. Traditional pharmacological interventions focus on glycemic control, blood pressure management, and RAAS blockade. Antidiabetic agents such as metformin and SGLT2 inhibitors have shown potential renoprotective effects. Furthermore, ACEIs and ARBs help reduce proteinuria and slow renal dysfunction progression.^[33]^ Natural products, including polyphenols, flavonoids, and saponins derived from plants, exhibit antioxidant, anti-inflammatory, and antifibrotic properties. These compounds may attenuate renal injury and preserve renal function, though further research is needed to explore their therapeutic potential.^[34]^

Dietary regulation plays a crucial role in the treatment of DN. Proper nutritional management aims to control blood glucose levels, blood pressure, and lipid profile. Recommendations include a balanced diet with controlled caloric intake, reduced sodium and protein intake, and increased consumption of fruits, vegetables, and whole grains. Dietary counseling and personalized meal plans are essential components of diabetes management.^[35]^

Recent development in the prevention and treatment of DN include the use of SGLT2 inhibitors and GLP-1 receptor agonists.^[20]^ SGLT2 inhibitors have been shown to reduce the risk of renal failure and cardiovascular events in patients with DN. GLP-1 receptor agonists have been noted to improve glycemic control and decrease the risk of cardiovascular disease in patients with diabetes and DN.^[8]^ Table 1 displays some preventive measures for DN, with supporting evidence from recent studies.

**Table 1:** Preventive measures for diabetic nephropathy.

*Glycemic control*: Maintaining reasonable glycemic control is essential in preventing diabetic nephropathy. Several studies have shown that tight glycemic control can reduce the risk of developing microalbuminuria, an early sign of kidney damage in diabetes.^[36]^ Intensive glycemic control can also slow the progression of diabetic nephropathy and reduce the risk of end-stage renal disease.^[37]^ *Blood pressure control*: Controlling blood pressure is another important measure in preventing diabetic nephropathy. Reducing blood pressure can reduce the risk of developing microalbuminuria and slow the progression of diabetic nephropathy.^[38]^ The American Diabetes Association recommends a blood pressure goal of <130/80 mm Hg for patients with diabetes.^[22]^ *Lipid management*: Lipid management is also crucial in preventing diabetic nephropathy. Elevated cholesterol and triglyceride levels can increase the risk of kidney damage in diabetes. Statins, a class of lipid-lowering medications, have been shown to reduce the risk of albuminuria in patients with type 2 diabetes.^[39]^ *Smoking cessation*: Smoking is a known risk factor for diabetic nephropathy. Smoking cessation can reduce the risk of developing microalbuminuria and slow the progression of diabetic nephropathy.^[40]^ *Regular monitoring*: Regular monitoring of kidney function is essential in the prevention of diabetic nephropathy. Early detection and treatment of kidney damage can prevent or delay the progression of diabetic nephropathy. The American Diabetes Association recommends annual screening for microalbuminuria in patients with type 1 diabetes and those with type 2 diabetes who have had diabetes for more than five years.^[41]^ *Lifestyle modifications*: Lifestyle modifications such as regular physical activity, healthy eating habits, and weight management can also help prevent diabetic nephropathy. Exercise has been shown to improve kidney function and reduce the risk of kidney damage in diabetes.^[42]^ A healthy diet, low in salt and saturated fat, can help control blood pressure and lipid levels.^[43]^ *Medications*: Medications such as angiotensin-converting enzyme inhibitors and angiotensin receptor blockers are recommended for managing diabetic nephropathy. These medications can reduce proteinuria, slow the progression of diabetic nephropathy, and reduce the risk of end-stage renal disease.^[44]^ *Education and awareness*: Education and awareness are crucial in preventing diabetic nephropathy. Patients with diabetes should be educated about the importance of glycemic control, blood pressure control, and lipid management. They should also be aware of the risk factors for diabetic nephropathy and the importance of regularly monitoring kidney function.

### 3.7. Comorbidities in DN

DN is a frequently occurring complication of diabetes, affecting approximately 30% of diabetic patients.^[8]^ It is characterized by structural and functional abnormalities of the kidneys, leading to a progressive decline in renal function.^[36]^ Patients with DN often have other comorbidities, which can further complicate the management of their condition. This article will discuss the comorbidities associated with DN and recent advances in their prevention and treatment.

#### 3.7.1. Hypertension.

Hypertension is a common comorbidity in patients with DN.^[45]^ It accelerates the decline in renal function and increases the risk of cardiovascular disease.^[46]^ Tight blood pressure control is essential in the management of DN. ACEIs and ARBs are the preferred agents for blood pressure control in patients with DN.^[22]^

#### 3.7.2. Dyslipidemia.

Dyslipidemia is also common in patients with DN.^[47]^ Elevated levels of triglycerides, low-density lipoprotein cholesterol, and total cholesterol are associated with an increased risk of cardiovascular disease and progression of DN.^[48]^ Statins are the preferred agents for the management of dyslipidemia in patients with DN.^[49]^

#### 3.7.3. Anemia.

Anemia is a common complication of DN, with a prevalence of up to 40%.^[50]^ It is mainly due to decreased erythropoietin production by the kidneys. Treatment with erythropoietin-stimulating agents can improve anemia and quality of life in patients with DN.^[51]^

#### 3.7.4. Neuropathy.

Diabetic neuropathy is a common complication of diabetes, affecting up to 50% of patients.^[52]^ It can lead to pain, sensory loss, and motor weakness. In patients with DN, neuropathy can further complicate the management of the condition. Tight glycemic control, analgesics, and antidepressants are the mainstays of treatment for diabetic neuropathy.^[53]^

#### 3.7.5. Obesity.

Obesity is a risk factor for developing and progressing DN.^[12]^ Weight loss through lifestyle modifications and bariatric surgery has been shown to improve renal function in obese patients with DN.^[54]^

#### 3.7.6. Depression.

Depression is common in patients with diabetes and has been shown to increase the risk of complications, including DN.^[55]^ The use of antidepressants and psychotherapy can improve depression symptoms and may also benefit the management of DN.^[56,57]^

## 4. Conclusion

Over the past years, significant advances have been made in preventing and treating DN, a complication of diabetes that affects the kidneys. This progress has been achieved through a better understanding of the underlying mechanisms of the disease, improved diagnostic tools, and the development of new therapeutic strategies. Preventive measures such as tight control of blood glucose, blood pressure, and lipid levels have decreased the risk of developing DN. Furthermore, making lifestyle adjustments such as engaging in regular physical activity and adopting a nutritious diet can also aid in preventing the development and progression of the disease. Several pharmacological interventions have also been developed for the treatment of DN. Angiotensin-converting enzyme inhibitors (ACE inhibitors) and ARBs are commonly used to reduce blood pressure and proteinuria in patients with DN. Other promising therapies include sodium-glucose cotransporter 2 (SGLT2) inhibitors, which have been shown to improve kidney function and reduce the risk of cardiovascular events in patients with diabetes. In addition to traditional pharmacological interventions, emerging therapies such as stem cell therapy, gene therapy, and immunotherapy hold promise for the future treatment of DN.

Overall, recent advances in the prevention and treatment of DN have provided new insights into managing this challenging complication of diabetes. With continued research and development, these advances are expected to lead to improved outcomes for patients with DN and a reduction in the burden of this disease on individuals and society.

## Author contributions

**Conceptualization:** Chukwuka Elendu, Boluwatife I. Adeyemo, Eunice K. Omeludike.

**Data curation:** Chukwuka Elendu, Boluwatife I. Adeyemo, Eunice K. Omeludike.

**Formal analysis:** Chukwuka Elendu, Babajide Obidigbo.

**Funding acquisition:** Chukwuka Elendu.

**Investigation:** Chukwuka Elendu.

**Methodology:** Chukwuka Elendu, Kemeasoudei D. J. Fiemotongha.

**Project administration:** Chukwuka Elendu, Blessing N. Bassey, Eunice K. Omeludike, Babajide Obidigbo.

**Resources:** Chukwuka Elendu.

**Software:** Chukwuka Elendu, Eunice K. Omeludike.

**Supervision:** Chukwuka Elendu, Blessing N. Bassey.

**Validation:** Chukwuka Elendu, Minichimso John Okah, Babajide Obidigbo.

**Visualization:** Chukwuka Elendu, Minichimso John Okah, Babajide Obidigbo.

**Writing – original draft:** Chukwuka Elendu.

**Writing – review & editing:** Chukwuka Elendu, Minichimso John Okah, Kemeasoudei D. J. Fiemotongha, Eunice K. Omeludike, Babajide Obidigbo.
